# Pathways Improving Compliance with Preventive Behaviors during the Remission Period of the COVID-19 Pandemic

**DOI:** 10.3390/ijerph18073512

**Published:** 2021-03-28

**Authors:** Jingjing Wang, Nanyue Rao, Buxin Han

**Affiliations:** 1 CAS Key Lab of Mental Health, Institute of Psychology, Beijing 100101, China; wangjj@psych.ac.cn (J.W.); raony@psych.ac.cn (N.R.); 2 Department of Psychology, University of Chinese Academy of Sciences, Beijing 100049, China

**Keywords:** COVID-19, risk perception, compliance with preventive behaviors, fear, anxiety, political trust, government dependency, dispositional optimism

## Abstract

The COVID-19 pandemic poses a significant threat to people’s lives. Compliance with preventive behaviors, recommended by public health authorities, is essential for infection control. In the remission stage, one year after the initial COVID-19 outbreak in China, we advanced a moderated parallel mediation model of the link between risk perception and compliance with preventive behaviors as well as a serial mediation model of the link between optimism and compliance with preventive behaviors, explaining the roles of various psychosocial factors in these associations. In January 2021, 200 participants under 50 years of age, located in 80 Chinese cities, participated in an online survey assessing risk perception, compliance with preventive behaviors, fear, anxiety, political trust, government dependency, and dispositional optimism. The results showed that the effect of risk perception on compliance with preventive behaviors was mediated by political trust and fear, and was moderated by government dependency. Anxiety and fear serially mediated the effect of optimism on compliance with preventive behaviors. Our study provided implications for future research to reduce negative emotions, strengthen confidence in the government, and sustain moderate government dependency accompanied by individual self-efficacy.

## 1. Introduction

Coronavirus Disease-2019 (COVID-19), a serious new infectious disease, was officially identified by the World Health Organization (WHO) as a pandemic in March 2020 [1]. To realize the goal of eliminating the COVID-19 pandemic, many governments worldwide have enacted draconian measures to prevent further spread. The National Health Commission of the People’s Republic of China issued COVID-19 Prevention and Treatment Guidelines for personal preventive health behavior [2]. Citizens’ adherence to guidelines for preventive behaviors recommended by the government is particularly important at all times and can be impacted by a cluster of cognitive and psychosocial factors.

Since the COVID-19 outbreak began in December 2019, the pandemic has been a threat to mental health in China. With the preliminary remission of the pandemic, from mid-February to April 2020, literature has reported several changes in the psychological health conditions of Chinese participants. During the preliminary remission period, Zhu et al. [3] found that participants had lower Symptom Check List 90 (SCL-90) scores than when the outbreak was more active. In other studies, fear was found to decline significantly, while depression and anxiety had significantly increased during the second survey conducted at a different time point [4,5]. However, another longitudinal study contradicted these findings, showing no significant changes in depression and anxiety over time [6]. Results from a study conducted in the Netherlands suggested a significant decline in the prevalence of anxiety and depression after June 2020, compared with data collected in June and March 2020 [7]. Thus, based on the studies above, mental health outcomes can differ depending on the timeframe, and different populations may lead to various conclusions. Therefore, one year following the initial COVID-19 outbreak in China, cognitive and psychosocial factors related to compliance with preventive behaviors, and the potential underlying mechanisms, should be reconsidered.

Several meta-analyses have shown that perception risk probability as a cognitive risk factor is a significant predictor of various health behaviors, which suggests that risk perceptions are important for health-related behavior [8,9,10,11]. In the case of the COVID-19 pandemic, research data collected starting at the beginning of the outbreak through April 1, 2020, have shown a positive relationship between perceived risk and compliance with COVID-19 preventive behaviors [12,13,14]. However, previous research has suggested that variations in risk perceptions depend on contextual factors [15,16], such as specific timeframes. Accordingly, there is a need to investigate the relationship between risk perception and health behaviors at different times within the COVID-19 outbreak. To date, the relationship between risk perception and preventive behaviors as well as the possible mechanism underlying this relationship have yet to be clarified during the remission period one year after the initial outbreak of the COVID-19 pandemic.

From among a range of psychosocial factors, previous research has demonstrated that risk perception is positively related with fear [14,17,18], and fear is significantly positively associated with preventive behaviors [14,19,20]. However, research has also shown contrasting evidence on the positive effects of fear [21]. Thus, the role of fear in the relationship between risk perception and preventive behaviors has not yet been revealed.

In terms of a political backdrop, the social exchange theory maintains that the costs arising from a social exchange relationship between the government and citizens influence political trust [22,23]. In regard to the COVID-19 pandemic, the Chinese government took unconventional and powerful measures for infection control; in return, they received political trust from citizens who are supportive of and satisfied with these powerful measures. Nunkoo and Ramkissoon’s study [24] holds that local residents’ costs perception of tourism negatively affected trust in government. In fact, except for tourism areas, people with low levels of food safety risk perception showed high levels of trust in the government [25]. To date, the influence of risk perception on political trust has yet to be clarified based on the context of the COVID-19 pandemic. Considering the special nature of the COVID-19 pandemic, the association between political trust and preventive behaviors has also been a key factor in previous research. Government trust was shown to be positively related to compliance with preventive behaviors [21,26,27,28]; however, there have been conflicting findings [29,30].

Based on the current circumstances in China, researchers have proposed a concept of government dependency, which refers to the interdependency between the citizens and their government—a supportive psychological orientation of the public toward their political system. Such psychological systems are relatively stable and persistent. Findings have shown that such government dependency predicts political trust in that increases in each level of dependence on the central government led to an increased probability of political trust by 107.09% [31]. Based on the above discussion, we intend to establish a moderated parallel mediation conceptual model to investigate the effect of risk perception on compliance with preventive behaviors mediated by political trust and fear and moderated by government dependency.

Furthermore, results on the relationships between anxiety and preventive behaviors and between fear and preventive behaviors have also been mixed [14,19,20,21,32,33,34,35], while dispositional optimism has been found to be positively related to preventive behaviors and negatively associated with fear [36]. Additionally, health anxiety was a predictor of fear of death from COVID-19 [37]. In view of the above-mentioned reasons, we intend to establish a serial mediation model between optimism and compliance with preventive behaviors mediated by anxiety and then fear.

In general, based on the above mixed findings regarding the association among cognitive and psychosocial factors and compliance with preventive behaviors and on the view that the pandemic situation changes over time in relation to mental health, we aimed to use a cross-sectional survey one year after the initial COVID-19 outbreak to explore the relationships among a range of cognitive and psychosocial factors and compliance with preventive behaviors as well as the underlying mechanisms relating to these variables.

## 2. Materials and Methods

### 2.1. Participants

Questionnaires were administered using “Credamo” (www.credamo.com, accessed on 9 January 2021), which is a reliable Chinese data collection platform, similar to Qualtrics, with quality control established through answer credit scores and historical adoption rate filtering. According to the Barrett’s recommendation for necessary and reasonable sample size [38], a total of 200 participants completed the questionnaire and were included in the data analysis. Participants were located in 80 Chinese cities (*M_age_* = 27.87, *SD* = 4.85; 54.5% male). Regarding marital status, 44.5% were unmarried and 55.5% were married. Further, 12% of participants had a monthly income below CNY 3000, 17% had an income between CNY 3001–5000, 30.5% had an income between CNY 5001−7000, 24.5% had an income between CNY 7001−9000, and 16% had an income above CNY 9000. Based on these data, 88% had an income above CNY 3000 (12% of participants had a monthly income below CNY 3000), which exceeds the average 2020 Chinese per capita monthly income (CNY 2682) [39].

### 2.2. Measures

All participants were required to provide socio-demographic data (age, gender, marital status, and income) and complete several questionnaires related to risk perception, compliance with COVID-19 prevention behavior, fears, anxiety, optimism, political trust, and government dependency.

#### 2.2.1. Risk Perception

We measured risk perception by adapting an item from Wen et al. [40], into a self-construed scale, namely, perceived risk probability (“I have a high risk of infection”), Participants rated this item on a 5-point Likert scale ranging from 0 (not at all) to 4 (very).

#### 2.2.2. Compliance with COVID-19 Prevention Behavior

We measured compliance with preventive behavior with a single question: “How often do you perform the following preventive measures for COVID-19?” Participants responses were scored on a 4-point Likert scale ranging from 1 (never) to 4 (often). The prevention behavior scale consisted of eight types of preventive behaviors, outlined by the COVID-19 Prevention and Treatment Guidelines for personal prevention issued by the National Health Commission of the People’s Republic of China [2]. These preventive behaviors included: frequent handwashing; wearing a mask; staying home; keeping the washroom clean; dining separately; cleaning, disinfection, and ventilation; maintaining social distance and covering coughs; and maintaining a healthy and regular life.

#### 2.2.3. Fear

We assessed fear using a two-item scale [20] rated on a 5-point Likert scale ranging from 0 (not at all) to 4 (very). These items were as follows: “The thought of the coronavirus makes me feel scared” and “I am afraid that someone in my family may get sick from the coronavirus”.

#### 2.2.4. Anxiety

We assessed anxiety using eight items from the Self-Rating Anxiety Scale (SAS) [40,41]. Example items included: “I feel calm and can sit still easily” and “I feel that everything is all right and nothing bad will happen.” Items were rated on a 4-point Likert scale ranging from 1 (some of the time) to 4 (most of the time).

#### 2.2.5. Optimism

We used the Life Orientation Test—Revised (LOT-R) to measure optimism with ten items adapted from previous studies [42,43]. Four of the ten items are filter items.

#### 2.2.6. Political Trust

Following Min et al. [21], we measured political trust with a two-item scale assessing trust in the central government and local government. Participants responded using a 5-point Likert scale ranging from 1 (completely distrust) to 5 (completely trust).

#### 2.2.7. Government Dependency

After first reviewing the relevant literature, we were unable to find any scale that explicitly measures government dependency related to the COVID-19 pandemic. Thus, referring to the political trust scale, we designed two items to measure political dependency on both the central government and local government. The items included “How dependent are you on the central government?” and “How dependent are you on the local government?”. Items were rated on a 5-point Likert scale ranging from 1 (completely independent) to 5 (completely dependent).

### 2.3. Data Analytic Strategy

The explanatory roles of fear, anxiety, optimism, political trust, and political dependency in the relationship between risk perception and compliance with preventive behaviors were tested. Descriptive statistics and bivariate correlations were performed using IBM SPSS 23.0 (IBM Corp., Armonk, NY, USA). Several simple path analysis models of observed variable were estimated using the ordinary least squares (OLS) method and the bootstrapping method, to estimate the indirect effect of risk perception on compliance with preventive behaviors via potential psychosocial variables, using the Hayes’ SPSS PROCESS macro [44,45]. Analysis of the differences in conditional indirect effects was conducted using Mplus 8.3 [46]. Specifically, the bootstrapping procedure, as a nonparametric resampling method, was used to test the significance of the total, direct, and indirect effects, as well as discrepancies in these effects at all levels of the potential moderator variables with 5000 bootstrap samples [47].

In accordance with previous studies [20,21], age, gender, marital status, and income were included as covariates in all analyses.

## 3. Results

Descriptive statistics and bivariate correlations are displayed in Table 1.

### 3.1. Moderated Parallel Mediation Analysis (Risk Perception to Preventive Behaviors)

**Figure 1:**
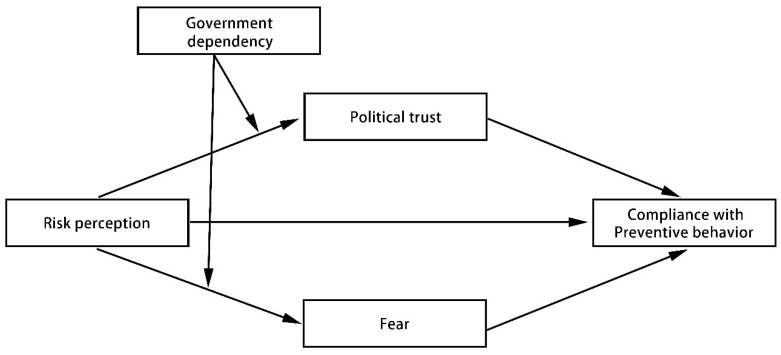
Conceptual model; the indirect effect of risk perception on compliance with preventive behaviors via political trust and fear, as moderated by government dependency.

To test our conceptual Model 1 (Figure 1, Table 2), we conducted moderated parallel mediation analyses controlling for a range of demographic variables using the SPSS PROCESS macro [45]. Using PROCESS Model 4, we found that the indirect effect of risk perception on compliance with preventive behaviors via political trust was significant (indirect effect: index = −0.0228, bootstrapped 95% CI = [−0.0698, −0.0008], accounting for 24.6% of the total indirect effect) and the indirect effect of risk perception on compliance with preventive behaviors via fear was also significant (indirect effect: index = −0.0698, bootstrapped 95% CI = [−0.1328, −0.0208], accounting for 75.3% of the total indirect effect). Using PROCESS Model 7, the conditional indirect effects were all significant under political trust (index = 0.0337, bootstrapped 95% CI = [0.0028, 0.0892]) and fear (index = −0.0296, bootstrapped 95% CI = [−0.0847, −0.0009]) as mediators.

**Figure 2:**
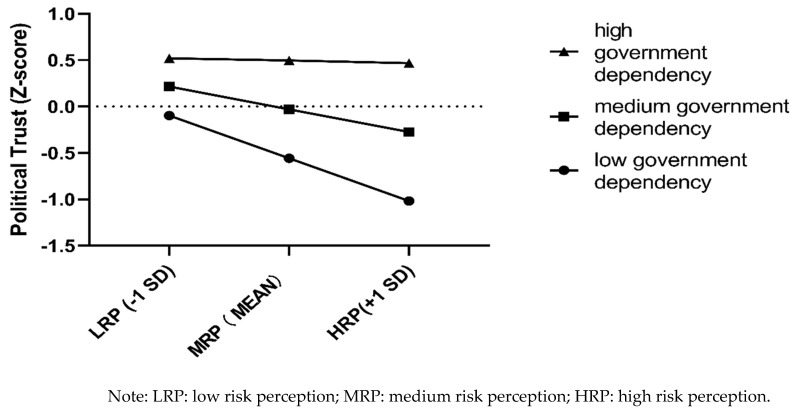
The interaction effect of risk perception and government dependency on political trust.

**Figure 3:**
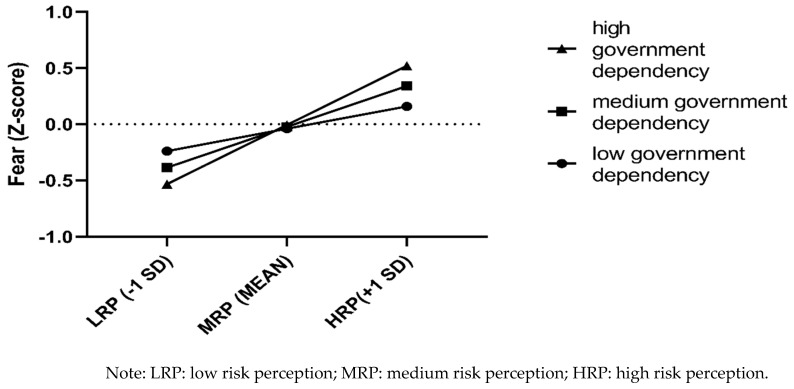
The interaction effect of risk perception and government dependency on fear.

In the high government dependency group, the indirect effect via political trust was not statistically significant, and the model was completely mediated by fear. In the low government dependency group, the indirect effect via fear was not statistically significant, and the model was completely mediated by political trust. In the medium government dependency group, the indirect effect of both mediators was significant; thus, the model was completely mediated by political trust and fear. Thus, the effect of risk perception on compliance with preventive behaviors was mediated by political trust when participants depended on the government at a low to medium level (Figure 2), while it was mediated by fear when participants depended on the government at a medium to high level (Figure 3). The difference between the conditional indirect effects was significant (index = 0.055, bootstrapped 95% CI = [0.009, 0.131]), as analyzed using Mplus 8.3. This finding suggests that the moderated mediation effect of government dependency is larger via political trust than via fear.

### 3.2. Serial Mediation Analysis (Optimism to Preventive Behaviors)

**Figure 4:**
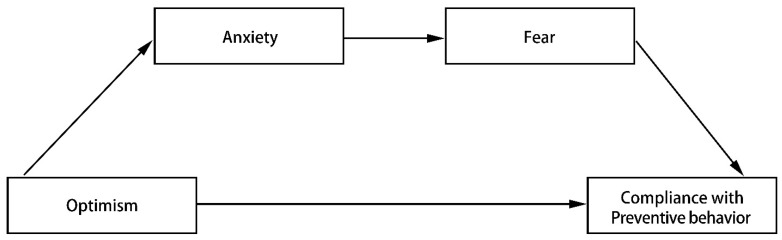
Conceptual model, the indirect effect of optimism on compliance with preventive behaviors via anxiety and then fear.

To test our conceptual Model 2 (Figure 4, Table 2), we conducted a serial mediation analysis controlling for a range of demographic variables. Using PROCESS Model 6, we found that only the indirect effect of optimism on compliance with preventive behaviors via anxiety and then fear was significant (indirect effect: index = 0.0452, bootstrapped 95% CI = [0.0184, 0.0863]). Thus, optimism predicted high compliance with preventive behaviors indirectly through lower levels of anxiety and fear.

## 4. Discussion

The current study makes theoretical contributions to the existing literature on the relationship among cognitive, psychosocial factors, and compliance with preventive behaviors [12,13,14,21,26,27,28,29,30]. There are several different paths to predict compliance with preventive behaviors for COVID-19. We advanced a moderated parallel mediation model of the link between risk perception and compliance with preventive behaviors and developed a serial mediation model between optimism and compliance with preventive behaviors mediated by anxiety and then fear. The present study examined the relationships among compliance with preventive behaviors, and a range of cognitive, psychosocial factors at the root level.

First, over the last few decades, the close association between risk perception and preventive behaviors has been recognized [8,9,10,11]. In the context of the COVID-19 pandemic, during the initial outbreak period, a positive direct relationship between perceived risk and compliance with COVID-19 preventive behaviors has also been empirically demonstrated [12,13,14]. However, as pointed out by Leventhal et al. [15], variations in risk perceptions depend on the specific timeframe, leading to the necessity of considering the relationship between these two variables and potential underlying mechanisms during the remission period one year after the initial COVID-19 outbreak.

Second, we did not find significant direct relationships between risk perception and compliance with preventive behaviors or between anxiety and compliance with preventive behaviors; however, a moderated parallel mediation was found between risk perception and compliance with preventive behaviors. The effect of risk perception on compliance with preventive behaviors was mediated by political trust and fear and moderated by government dependency. Lower risk perception was associated with higher political trust and lower fear in relation to COVID-19, which, in turn, were associated with higher compliance with preventive behaviors. These results are consistent with previous studies showing direct negative associations between political trust and the perceived threat of COVID-19 [48], as well as a positive relationship between political trust and compliance with preventive behaviors [21,28] and a positive association between risk perception and fear [14].

However, our findings were inconsistent with a previous study [20], which found a negative association between fear and preventive behaviors. This can possibly be explained by the cognitive resource theory and the mental protection of collectivism. In particular, resisting negative emotions depletes many cognitive resources for reducing preventive intention, and collectivism builds mental barriers against fear from threats [21,49,50,51]. In addition, the mediating effect of fear was found to be larger than political trust in this parallel mediation. This showed that the effect of risk perception on compliance with preventive behaviors in China was mainly mediated by the emotion of fear, as opposed to political trust, during the pandemic.

Notably, the parallel mediation between risk perception and compliance with preventive behaviors was moderated by government dependency. To our knowledge, this study was the first to investigate the explicit role of government dependency in health psychology. The effect of risk perception on compliance with preventive behaviors via political trust was not statistically significant in the high government dependency group; however, the effect of risk perception on compliance with preventive behaviors was mediated by fear. Nonetheless, for those people with weak dependence on the government for protection against COVID-19, the opposite may also be true.

High government dependency decreases the effect of risk perception on political trust. People in this group had relatively high scores for political trust irrespective of risk perception. For people in the low and medium government dependency groups, risk perception negatively predicted political trust. In contrast, low government dependency significantly decreases the effect of risk perception on fear. People in this group scored closer to the mean for fear among the low-, medium-, and high-risk perception groups. Thus, for people in the medium and high government dependency groups, risk perception positively predicted fear. The one boundary condition for the effect of risk perception on compliance with preventive behaviors through political trust and fear separately depends on different levels of government dependency. During the present remission period one year after the outbreak of the COVID-19 pandemic, those with moderate government dependency have the advantage of increased political trust combined with reduced fear, which contributes to the implementation of COVID-19 prevention measures. Government dependency and political trust have different functions in the relationship between risk perception and compliance with preventive behaviors, which further shows they are different constructs [31].

During the COVID-19 pandemic, the Chinese government took unconventional and powerful measures to reduce the spread of infection [52], such as mobilizing nationwide medical staff and products to support Wuhan, which further intensified the government dependency and political trust of Chinese citizens. Together with collectivism and the tradition of authority dependency [53], depending on the government to deal with a major situation is very common in China. However, a high level of government dependency may reduce individual self-efficacy for fighting the pandemic in daily life. Wong and Jensen [30] posited that it is necessary to prevent public complacency and responsibility relegation due to public trust in the government. Thus, we consider that maintaining moderate government dependency and making essential individual efforts would be more ideal.

Third, this study clarified another serial mediation path for predicting compliance with preventive behaviors. The results showed that the relationship between optimism and compliance with preventive behaviors was mediated by anxiety and then fear. However, the direct effect was not significant when controlling for a range of demographic variables, which is inconsistent with previous research [30]. This may be explained by the previous study not controlling for demographic factors. Our findings are consistent with previous studies on the association between optimism and less coronavirus-related anxiety, and the positive relationship between anxiety and fear [54,55,56,57]. The serial mediation model between optimism and compliance with preventive behavior suggested that the cognitive personality variable works through emotional factors to predict preventive behaviors, which again emphasized the essential role of negative emotions in influencing preventive behaviors. 

In addition to cognitive and psychosocial factors, the physical factor could be considered an important factor affecting preventive behavior during the pandemic, which should be investigated in the future. The possible underlying mechanism was through psychological factors. Previous studies showed that regular physical activity during the COVID-19 pandemic can help to reduce stress and negative emotions [58,59]. During the confinement period of the pandemic, indoor exercise is more suited to maintaining physical and mental health while protecting one from infection with COVID-19 [60]. For example, active videogames are a home-based activity and a highly suitable way to cope with negative emotions [61].

Our study does have some limitations that should be considered. First, our data were collected using a cross-sectional design, which limits causal interpretations. Therefore, a longitudinal study should be conducted to verify these results in the future. Second, the sample size was relatively small in the present study, which may have limited the generalizability of the conclusions.

## 5. Conclusions

The present study advanced a moderated parallel mediation model of the link between risk perception and compliance with preventive behaviors as well as a serial mediation model of the link between optimism and compliance with preventive behavior. This was the first time the role of government dependency in the relationship between risk perception and compliance with preventive behaviors was explicitly investigated. The present study suggested that, when risk perception decreased, a medium level of government dependency was beneficial for increasing preventive behaviors because of the combination of increased political trust and decreased fear. Furthermore, the effect of dispositional optimism on compliance with preventive behaviors was serially mediated by anxiety and fear, which emphasizes the influence of emotion on preventive behaviors. In the current remission period one year after the initial outbreak of the COVID-19 pandemic, much work is still needed to further reduce negative emotions and strengthen confidence in the government, to sustain moderate government dependency accompanied by individual self-efficacy.

## Figures and Tables

**Table 1:** Descriptive statistics and correlations.

Variables	M	SD	Correlations					
			1	2	3	4	5	6
1. Risk perception	1.24	0.99						
2. Preventive behaviors	29.78	2.15	−0.128					
3. Fear	4.89	2.03	0.384 **	−0.193 **				
4. Anxiety	14.93	4.95	0.286 **	−0.055	0.45 **			
5. Political trust	7.13	0.95	−0.154 *	0.155 *	−0.003	−0.09		
6. Political dependency	6.24	1.53	0.133	0.132	0.004	0.054	0.428 **	
7. Optimism	17.87	3.19	−0.143 *	0.115	−0.162 *	−0.425 **	0.087	0.111

Note: Demographic characteristics were included as controls: age, gender, marital status, income. *N* = 200, * *p* < 0.05. ** *p* < 0.01.

**Table 2:** Bootstrap moderated parallel mediation effect and serial mediation effect.

Model	Paths	Point Estimate	SE	Bootstrap 95% CI	
				Lower	Upper
Model 1 Moderated parallel mediation analysis	Indirect effect				
	Risk perception**→**political trust**→**preventive behaviors	−0.0228	0.0168	−0.0698	−0.0008
	Risk perception**→**fear**→**preventive behaviors	−0.0698	0.0285	−0.1328	−0.0208
	Conditional indirect				
	Risk perception→political trust→preventive behaviors moderated by political dependency	0.0337	0.0214	0.0028	0.0892
	Risk perception→fear→preventive behaviors moderated by political dependency	−0.0296	0.0203	−0.0847	−0.0009
	Contrasts				
	Moderated mediation by political trust vs. by fear	0.055	0.03	0.009	0.131
Model 2					
Serial mediation analysis	Indirect effect				
	Optimism→anxiety→fear→preventive behaviors	0.0452	0.0167	0.0184	0.0863
	Total effect	0.1144	0.0716	−0.0268	0.2556
	Direct effect	0.1236	0.0782	−0.0307	0.2779

## Data Availability

The data presented in this study are available on request from the corresponding author.
